# Oscillating US Department of Defense policies and medical record documentation of gender dysphoria in service members: an observational time-series analysis

**DOI:** 10.1186/s12913-024-11731-4

**Published:** 2024-10-23

**Authors:** Krista B. Highland, David A. Klein, Sydney Rogers, Alexander G. Velosky, Christina Roberts, Noelle S. Larson

**Affiliations:** 1 Department of Anesthesiology, Uniformed Services University, 4301 Jones Bridge Road, Bethesda, MD 20814 USA; 2 Department of Family Medicine, David Grant Medical Center, Travis Air Force Base, CA USA; 3 Department of Family Medicine, Uniformed Services University, Bethesda, MD USA; 4 Department of Pediatrics, Uniformed Services University, Bethesda, MD USA; 5 School of Medicine, Uniformed Services University, Bethesda, MD USA; 6 Henry M. Jackson Foundation for the Advancement of Military Medicine, Inc, Bethesda, MD USA; 7 Enterprise Intelligence and Data Solutions (EIDS) program office, Program Executive Office, Defense Healthcare Management Systems (PEO DHMS), San Antonio, TX USA; 8 Division of Adolescent Medicine, Department of Pediatrics, Icahn School of Medicine at Mount Sinai, New York City, NY USA; 9 Department of Pediatrics, Walter Reed National Military Medical Center, Bethesda, MD USA

**Keywords:** Policy, Gender dysphoria, Gender-affirming healthcare, Patient disclosure

## Abstract

**Background:**

United States military policies regarding service by transgender service members have shifted several times within the past decade. The relationships between policy changes and electronic health record documentation of gender dysphoria, a current and historic policy requisite for gender affirming care receipt, in active duty service members remain unknown.

**Methods:**

Bayesian estimator of abrupt change, seasonality, and trend models identified changepoints in the proportion of service members who had new and then historical medical record documentation consistent with gender dysphoria from January 2015 to August 2022. Changepoints were evaluated as they related to salient military policy-related events.

**Results:**

Approximately 3,853 active duty and activated National Guard or Reserve service members received a documented diagnosis corresponding to gender dysphoria from January 2015 to August 2022. Four significant changepoints were identified across both time series. Salient historical events that occurred during the changepoint periods were identified for contextualization.

**Conclusions:**

Clinical documentation of gender dysphoria oscillated with changes to policies and public statements by government leaders, which may in turn, impact military recruitment and retention. This study highlights the need for equitable policies that optimize the strength of a diverse military force. Equity-oriented monitoring is needed to continually examine the impact of military service policies on readiness and retention to support actionable, data-driven improvements to policies and their implementation.

## Background

Transgender (trans) people have experienced and are experiencing significant barriers to active duty military service within the United States (US) [1–5]. Policies impacting Service eligibility and access to medically-necessary, equitable healthcare through the US Military Health System have oscillated widely since June 30, 2016, when trans people were first permitted to serve openly in uniform [2]. Restrictions were informally and then formally reimposed between 2017 and 2019 [1, 2]. Interim restrictions were formally lifted in April 2021 [6]. However, lack of clear implementation guidance, significant variability in administrative processes, and delays in system-wide standardization of healthcare access pathways led to persistent barriers to equitable medical readiness of trans service members.

The impact of inconsistent policy protections on service member disclosure to medical clinicians and clinician documentation of diagnoses reflective of gender dysphoria in the medical record, a policy-based requirement to receiving gender-affirming medical care, has not been systematically evaluated. Knowledge of barriers to disclosure in healthcare settings is essential to understanding access to and effectiveness of healthcare services to optimize military readiness. The present study aims to describe changes in the number of service members who received documentation of diagnoses reflective of gender dysphoria in their medical records in the US Military Health System, within the context of US Military policies, from January 2015 to August 2022. Analyses described overall monthly frequencies and proportions of active duty service members with newly and previously documented diagnoses reflective of gender dysphoria as policies changed, as well as variation by Service branch (Air Force, Army, Marine Corps, Navy), to support a data-driven approach to ongoing development of personnel and healthcare services policy.

## Methods

### Study design and record selection

This observational study was determined to be exempt from institutional review board review in accordance with (IAW) 32 Code of Federal Regulation 219.104(d), category [4] by the Determination Official at the Walter Reed National Military Medical Center Department of Research Program. Administrative permissions were required to access raw data. To access the raw data, a signatory of the US Defense Health Agency granted a Data Sharing Agreement to access the raw data within the Military Health System Information Platform after the exempt determination was obtained. Per the requirements set forth by the Data Sharing Agreement, all raw data was de-identified before analysis. The data utilized claims data extracted from the Military Health System Data Repository covering the period from January 2015 to August 2022. Records were selected for inclusion if the patient’s beneficiary category was listed as a service member on active duty, to include activated National Guard or Reserve. Selected records were only included if the Service branch was recorded as Air Force, Army, Marine Corps, or Navy. Other Service branches were not included due to low sample sizes. Lastly, selected records were only included if the active duty service member received a documented diagnosis reflective of gender dysphoria; these diagnoses were identified using previously validated International Classification of Diseases (ICD)-9 and − 10 codes [7].

### Terminology use

The ICD-9 and − 10 codes that were in use during the study period referenced terms like “gender identity disorder,” which are outdated and inaccurate. These terms have since been removed in the most recent ICD-11 and gender incongruence was added as a diagnosis in the chapter focused on sexual health [8]. The present study refers to diagnoses as “corresponding to” or “reflective of” gender dysphoria, as this diagnosis was required to receive gender-affirming medical care (e.g., hormone therapy, surgery) per military policy [6].

We report monthly frequencies and proportions of service members who (1) received a newly documented diagnosis reflective of gender dysphoria and (2) had historically received a diagnosis reflective of gender dysphoria and remained on active duty. We intentionally do not use the terms incidence and prevalence, as these terms are typically applied in reference to disease states, whereas receipt of a diagnosis corresponding to gender dysphoria should not be discussed through a pathologizing lens. Not all trans service members will seek gender-affirming healthcare during active duty service. Therefore, the inclusion criteria in the present study should not be used to estimate the representation of trans people in the US service member population.

Administrative records of service members include a variable that is referred to, per policy [6], as a “gender marker.” Currently, only two administrative gender markers are available to personnel and medical records, male or female. These administrative gender markers exclude non-binary and gender diverse service members who would, if options were available, use another gender marker. Moreover, the term “gender marker” is inaccurate for trans service members for whom the administrative gender marker reflects their sex assigned at birth and not their gender. For the purposes of the present study, the first administrative gender marker was identified for descriptive purposes as a proxy for sex assigned at birth.

### Analytic plan

Once service members received a documented diagnosis corresponding to gender dysphoria, they remained in the cohort with a historical diagnosis reflective of gender dysphoria through their most recent month of recorded active duty status. Data were cleaned using the *dplyr* [9], *janitor* [10], and *vroom* [11] R packages and visualized using *ggplot2* R Package [12]. Accessible colors were selected using the Adobe Color Blind Safe Accessibility Tool. Characteristics of the overall sample and by first administrative gender marker were aggregated and displayed in a table using the *compareGroups* R package [13]. To contextualize the active duty sample with the overall active duty population, data from the 2017–2022 Military OneSource Demographics Profile [14] were aggregated and averaged across the available six years.

Monthly frequencies and proportions of total number of active duty service members with new and previously documented diagnoses corresponding to gender dysphoria were calculated. Monthly proportions were then evaluated using Bayesian estimator of abrupt change, seasonality, and trend (BEAST) models. BEAST models enabled changepoint detection, while accounting for seasonality and trends inherent to time series data [15]. Changepoint detection was completed using the *Rbeast* R package [15], to include iteration of 1,000 Markov chain Monte Carlo (MCMC) samplings across each model. Each changepoint was provided a probability of a true changepoint, as well as the 95% credibility interval (95% CI). The changepoints were then contextualized within salient events for descriptive purposes.

## Results

**Fig. 1:**
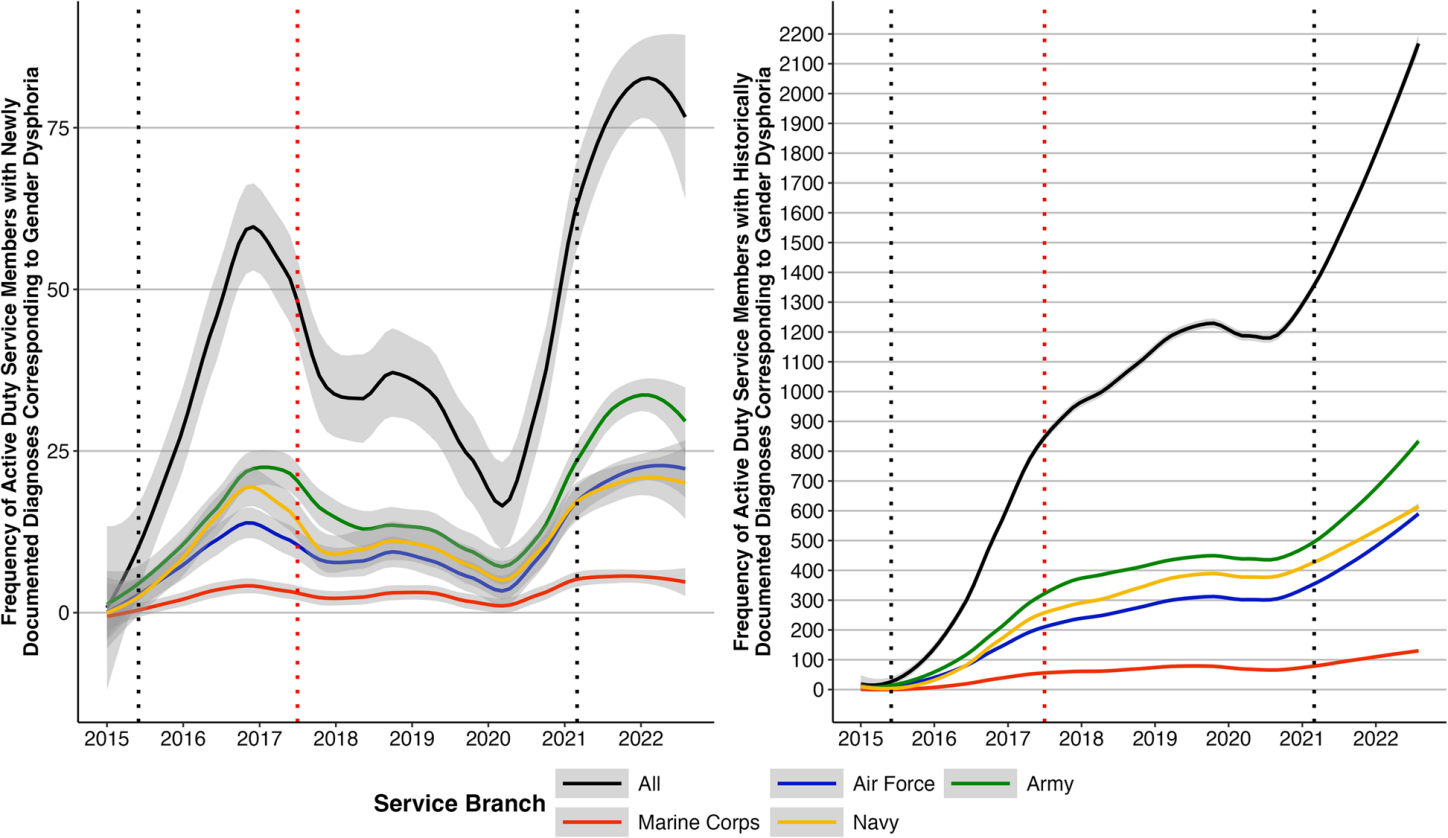
Monthly frequencies of service members with newly (left) and historically (right) documented diagnoses corresponding to gender dysphoria by Service and overall

Approximately 3,853 active duty and activated National Guard or Reserve service members received a documented diagnosis corresponding to gender dysphoria between January 2015 to August 2022 (Fig. 1). Demographic information of service members included in the analysis, as well as by first administrative gender marker are reported in Table 1. Most of the sample (93.4%) were active duty service members. As described in Table 2, there was a higher proportion of active duty service members in this sample, relative to the 2017–2022 overall active duty service member population, with a first administrative binary gender marker of female (54.9% and 17.0%, respectively), race recorded as white (73.8% and 68.8%, respectively), age of 17–25 years (62.5% and 44.9%, respectively), and rank of junior enlisted (66.3% and 43.1%, respectively). There was a lower proportion of active duty service members in this sample, relative to the 2017–2022 overall active duty service member population, who were Latina/e/o (14.5% and 16.9%, respectively) and in the Marine Corps (7.4% and 13.8%, respectively).

**Table 1:** Demographic information of service members whose medical record contained a documented diagnosis corresponding to gender dysphoria from January 2015 to August 2022 overall and by first administrative binary gender marker

		First Administrative Binary Gender Marker	
**Variable**	***Full Sample*** *(N = 3853)*	***Male*** *(n = 1753*,*** 45.5%)***	***Female*** *(n = 2100*,*** 54.4%)***
Beneficiary Category, n (%)			
Active Duty	3598 (93.4%)	1624 (92.6%)	1974 (94.0%)
National Guard or Reserve on Active Duty	255 (6.6%)	129 (7.4%)	126 (6.0%)
Race, n (%)			
American Indian & Alaskan Native	47 (1.2%)	22 (1.3%)	25 (1.2%)
Asian & Pacific Islander	191 (5.0%)	92 (5.2%)	99 (4.7%)
Black	636 (16.5%)	446 (25.4%)	190 (9.0%)
More than one race reported	32 (0.8%)	14 (0.8%)	18 (0.9%)
Another and Unknown	94 (2.4%)	52 (3.0%)	42 (2.0%)
White	2853 (74.0%)	1127 (64.3%)	1726 (82.2%)
Latina/e/o, n (%)	550 (14.3%)	298 (17.0%)	252 (12.0%)
Age Group, n (%)			
Age 17–25	2456 (63.7%)	1197 (68.3%)	1259 (60.0%)
Age 26–30	799 (20.7%)	366 (20.9%)	433 (20.6%)
Age 31–35	329 (8.5%)	122 (7.0%)	207 (9.9%)
Age 36–40	159 (4.1%)	37 (2.1%)	122 (5.8%)
Age 41 and older	110 (2.9%)	31 (1.8%)	79 (3.8%)
Service			
Army	1526 (39.6%)	724 (41.3%)	802 (38.2%)
Air Force	970 (25.2%)	358 (20.4%)	612 (29.1%)
Marine Corps	273 (7.1%)	130 (7.4%)	143 (6.8%)
Navy	1084 (28.1%)	541 (30.9%)	543 (25.9%)
First Recorded Rank			
Junior Enlisted (E1-E4)	2480 (64.4%)	1202 (68.6%)	1278 (60.9%)
Senior Enlisted (E4-E9)	1034 (26.8%)	411 (23.4%)	623 (29.7%)
Cadet and Junior Officer (O1-O3)	182 (4.7%)	86 (4.9%)	96 (4.6%)
Senior Officer (O4+)	59 (1.5%)	15 (0.9%)	44 (2.1%)
Warrant Officer or Another Rank	32 (0.8%)	14 (0.8%)	18 (0.9%)
Missing	66 (1.7%)	25 (1.4%)	41 (2.0%)

Due to rounding, columns may not add up to 100%. All demographic information corresponded to enrollment information at first documentation for a diagnosis reflective of gender dysphoria. The first administrative binary gender marker is what is recorded in personnel and medical records and serves as a proxy for sex assigned at birth

**Table 2:** Demographic information of active duty service members* whose medical record contained a documented diagnosis corresponding to gender dysphoria from January 2015 to August 2022 and Department of Defense (DoD) active duty service members overall from 2017–2022**

Variable	Active Duty Sample (*n* = 3571)	DoD Active Duty Population**
First Administrative Binary Gender Marker, n (%)		
Male	1611 (45.1%)	83.0%
Female	1960 (54.9%)	17.0%
Race, n (%)		
American Indian & Alaskan Native	46 (1.3%)	1.1%
Asian & Pacific Islander	183 (5.1%)	6.0%
Black	591 (16.5%)	17.2%
^More than one race reported	31 (0.9%)	3.0%
Another and Unknown	85 (2.4%)	3.9%
White	2635 (73.8%)	68.8%
Latina/e/o, n (%)	518 (14.5%)	16.9%
Age Group, n (%)		
Age 17–25	2327 (65.2%)	44.9%
Age 26–30	746 (20.9%)	21.3%
Age 31–35	287 (8.0%)	15.0%
Age 36–40	134 (3.8%)	10.8%
Age 41 and older	77 (2.2%)	8.0%
Service		
Army	1338 (37.5%)	36.1%
Air Force	920 (25.8%)	24.7%
Marine Corps	264 (7.4%)	13.8%
Navy	1049 (29.4%)	25.4%
First Recorded Rank		
Junior Enlisted (E1-E4)	2367 (66.3%)	43.1%
Senior Enlisted (E4-E9)	928 (26.0%)	39.2%
Junior Officer (O1-O3)	156 (4.4%)	10%
Senior Officer (O4+)	46 (1.3%)	6.4%
Warrant Officer or Another Rank	14 (0.4%)	1.4%
Missing	60 (1.7%)	0%

*27 service members were excluded due to having ranks that were not included in the Military OneSource Demographics Profile data (e.g., cadets)

**DoD active duty (not National Guard or Reserve) population statistics from 2017–2022 reported in the right column based on Military OneSource Demographics Profile data (https://www.militaryonesource.mil/data-research-and-statistics/military-community-demographics/)

^The Army does not report this category

**Fig. 2:**
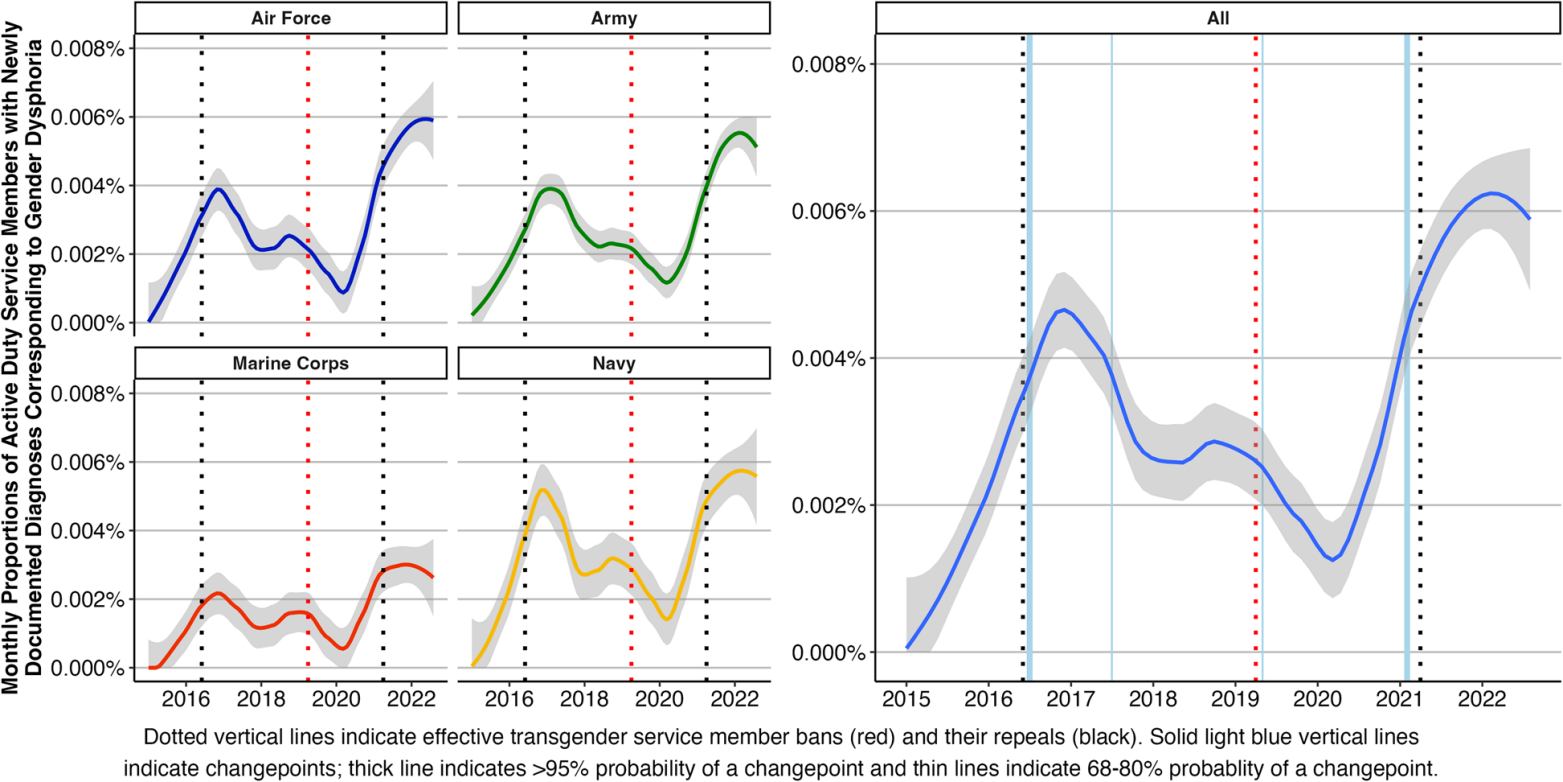
Monthly proportion of service members with newly documented diagnoses corresponding to gender dysphoria by Service (left) and overall with changepoints (right)

**Fig. 3:**
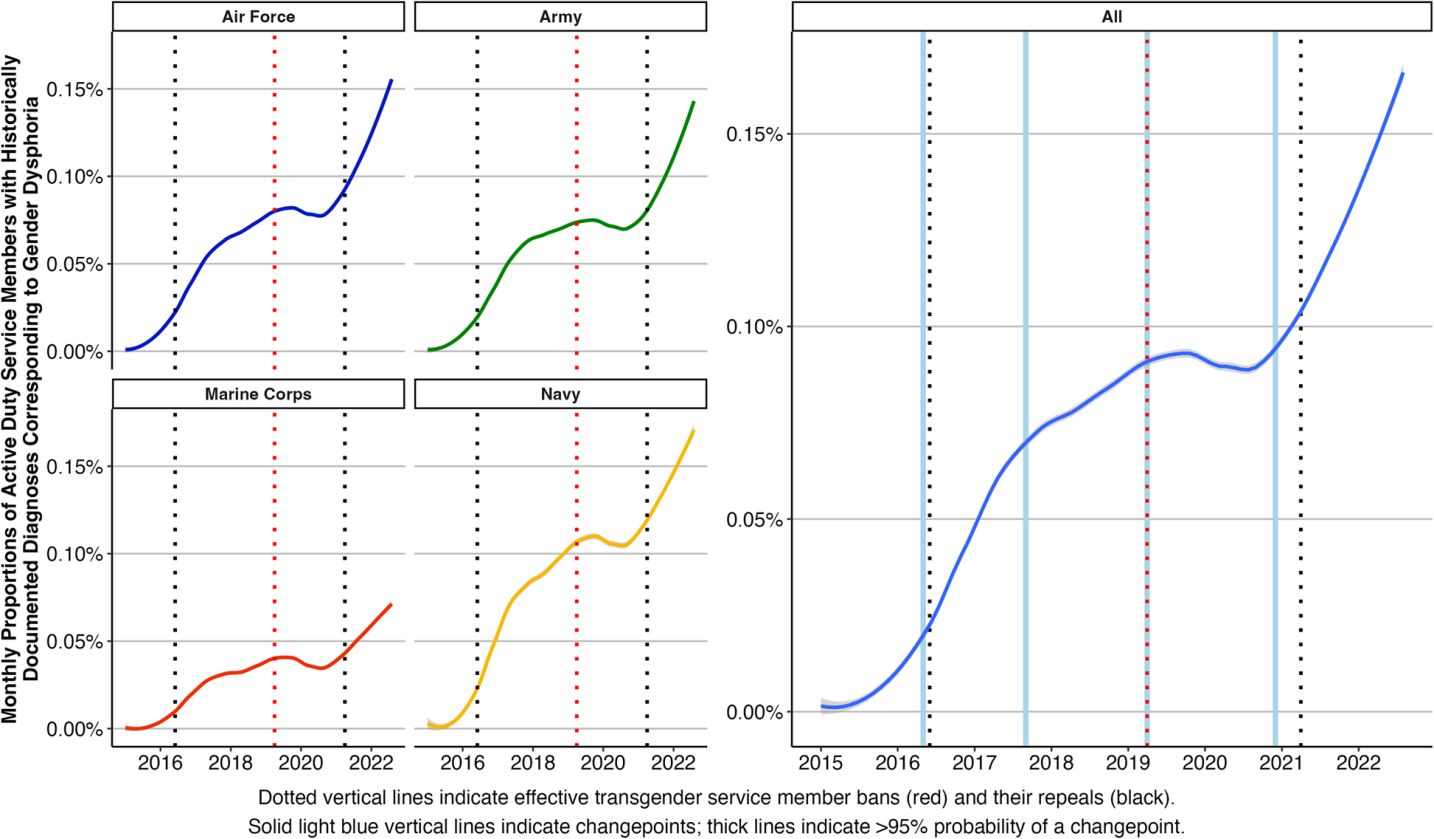
Monthly proportion of service members with historically documented diagnoses corresponding to gender dysphoria by Service (left) and overall with changepoints (right)

The number of service members with newly and historically documented diagnoses changed across time. Almost half (47.5%) of the MCMC samplings supported four trend changepoints in the proportion of service members with newly documented diagnoses, with the 10th and 90th percentile of samplings indicating four and six changepoints, respectively. Similarly, the majority of MCMC samplings (92.2%) supported four trend changepoints in the proportion of service members with historically documented diagnoses, with the 10th and 90th percentile both indicating four changepoints. As reported in Table 3, the 95% credible intervals of changepoints between the two models all had overlap, but probabilities varied between the models (67% to > 99.9%). The proportions of service members with newly and historically documented diagnoses corresponding to gender dysphoria by Service and overall are shown in Figs. 2 and 3, respectively.

**Table 3:** Changepoints (credible intervals) and their probabilities in the proportion of active duty service members with newly and historically documented diagnoses corresponding to gender dysphoria, January 2015 – August 2022

Newly Documented		Historically Documented		
Changepoint (Credible Interval)	Pr.	Changepoint (Credible Interval)	Pr.	Salient Events
July 2016 (June 2016 – September 2016)	98%	May 2016 (March 2016 – July 2016)	> 99%	*July 2015*: DoD Secretary Ash Carter releases a statement reflecting that policies related to open service are “outdated” and initiates study and action to develop new policy. *June 2016*: First Department of Defense policy allowed open service.
July 2017 (February 2017 – April 2018)	68%	September 2017 (May 2017 – October 2017)	> 99%	*July 2017*: Presidential tweet announced plan to reinstate bar to active duty service for most trans people; legal delays to enacting. *March 2018*: Secretary Mathis’s plan regarding service accepted by President. Plan challenged and went to Supreme Court.
May 2019 (November 2018 – October 2019)	80%	April 2019 (February 2019 – September 2019)	96%	*January 2019*: Supreme Court indicates the plan can go into effect. *April 2019*: DTM-19-004 again barred most open service.
February 2021 (January 2021 – March 2021)	> 99%	December 2020 (October 2020 – February 2021)	> 99%	*January 2021*: EO 14,004 directed removal of bar to open service. *April 2021*: Revision of DoDI again allowed open service. Service-specific policies released over several months.

*Pr. *Probability, *DoD *Department of Defense, *DoDI *Department of Defense Instruction, *EO *Executive Order

## Discussion

The present findings demonstrate that the likelihood of documentation of diagnosis corresponding to gender dysphoria in the electronic health record was associated with stated leadership intentions to enact a ban and subsequent military policy changes regarding open service. For example, the results indicated changepoints that align with effective bans, their repeals, as well as a July 2017 presidential social media post indicating “the United States Government will not accept or allow trans individuals to serve in any capacity in the US Military.” Taken together, it is both the signaling of potential policy change through stated leadership intention and implemented policy change that could have had a measurable impact on such disclosures.

Without a documented diagnosis, service members are ineligible to receive gender-affirming healthcare services. Furthermore, official change of the administrative gender marker in military personnel records, *if* an option that reflects one’s gender is available (e.g., male or female), also requires attestation of a diagnosis of gender dysphoria in health records. When desired and received, gender-affirming services can optimize wellness, increase the ability to contribute maximally to the mission, reduce negative health outcomes, and enhance operational readiness [16, 17]. Thus, policy oscillations that impact disclosure to clinicians and documentation of a diagnosis reflective of gender dysphoria in the health record may have significant health justice and operational readiness equity implications.

Within the Intersectionality Research for Transgender Health Justice Framework [18], multiple systemic and institutional factors may contribute to the lack of safety for trans service members to seek gender-affirming healthcare. From a policy perspective, knowing that open service may be permitted today, but can quickly become prohibited tomorrow may lead to non-disclosure and lack of medically-necessary care receipt. Moreover, trans and gender diverse Active Component service members, relative to their cisgender peers, are more likely to experience past-year unwanted sexual contact (6% versus 2%), sexual harassment (27% versus 10%), and gender discrimination (10% versus 4%) per a 2021 DoD-wide survey [19]. As such, the overarching workplace environment may prevent disclosure to clinicians.

At the healthcare institution-level, several barriers may limit gender-affirming healthcare seeking. The requirement for a diagnosis of gender dysphoria to receive medically-necessary care can be a barrier in and of itself [20]. Then, to continue to receive care (e.g., exogenous sex steroid hormone therapy), the diagnosis of gender dysphoria must be documented, even if symptoms reflective of gender dysphoria are no longer present, as there is currently no option to code such routine care as preventative healthcare (e.g., Z-codes in the ICD, similar to contraception care) or use a code indicating the diagnosis as being in remission (e.g., similar to cancer-related ICD codes).

The US Military Health System electronic health record currently lacks standardized fields for the accurate capture and display of sex assigned at birth, gender, and how to refer to service members respectfully (e.g., names, pronouns). This structural limitation has significant implications for healthcare experiences, quality, receipt, and trust [21, 22]. Combined with the lack of inclusive administrative gender markers for personnel records (e.g., inclusion of non-binary gender markers), the lack of electronic health record structure, utility, and training could lead to inappropriate documentation that is then accessible to service members through their patient portals and is a form of preventable pathologization [21].

The ubiquity of discriminatory and stigmatizing experiences in healthcare settings is well documented [23, 24], including in the US Military Health System. In a survey study, 65% of active duty trans and gender diverse service members reported experiences of stigma in the healthcare setting [25]. Additionally, more than half of surveyed US Military Health System clinicians reported they would not consider prescribing gender-affirming hormone therapy to adult patients, even if provided additional education or had assistance from experienced clinicians [4]. Such findings indicate that service members may have limited access to clinicians who are appropriately trained and willing to provide inclusive healthcare, which in turn, may limit disclosure.

For trans service members who ultimately receive an administrative gender marker change in their personnel records, the need for preventative health screenings (e.g., cervical cancer, prostate cancer) can be obfuscated and (health) equity monitoring [26] can be significantly limited. An inclusive approach to documenting service members’ gender and sex assigned at birth accurately in personnel and medical record systems would aid in the ongoing evidence-based evaluation of pertinent outcome measures. Future health services research is needed to ensure trans service members receive equitable preventative health screenings.

While the proportion of service members who historically received a diagnosis reflective of gender dysphoria increased during the effective trans service member ban from April 12, 2019 to April 30, 2021, reflecting temporal trends in the US population [27], changepoints indicated these increases significantly slowed during this period. The present analysis did not isolate impacts on new accessions versus attrition, but results may reflect an adverse impact of restrictive policies on recruitment and retention of trans people, as has been previously published [3]. As such, Service-level differences in diagnosis documentation patterns across time could also be reflective of potential differences in Service climate, policies, personnel trainings, and other institutional factors. Further research is needed to understand service members’ experiences across different Services to identify institutional factors that could be improved to support readiness.

Institutional inequities, such as the oscillating policies regarding open service and the lack of inclusive and accurate documentation of gender and sex assigned at birth in personnel and medical records, prevent evidence-based programming to address structural insufficiencies that can negatively impact health, safety, wellbeing, readiness, and retention of trans service members. Reducing these inequities is paramount to actualizing the principles of a high-reliability healthcare organization [28, 29]. To reduce inequities, genuine inclusion of trans service members in policy and program development would both embody the principles of a high-reliability healthcare organization and enable optimized implementation and medical readiness. The present findings inform clinicians of the need to ensure their preparedness for providing high-quality, timely, and inclusive healthcare.

The longitudinal nature of the present data, the large sample size, and opportunity to examine the effect of multiple changes in policy during the study period are robust strengths; however, this study also has several limitations. In addition to the institutional factors described above, restrictions in collecting sexual orientation and gender data, lack of options to self-identify gender distinct from sex assigned at birth in administrative and healthcare records [30], and lack of inclusion of trans people in pertinent policy development, may further reduce the likelihood that a diagnosis corresponding to gender dysphoria would be documented. The present data should not be used to estimate the total number of trans and gender diverse service members. Documentation of a diagnosis reflective of gender dysphoria is not a sound, accurate, or ethical means of population estimation. Finally, our data did not assess regional differences in frequencies and proportions of diagnoses, which may be associated with broader factors at multiple socio-ecological levels that impact access to healthcare.

## Conclusions

This study highlights the need for consistent policies that support inclusion of trans and gender diverse service members and reduce barriers to accessing healthcare services, which optimize the strength of a diverse military force. Prior DoD-commissioned reports [31] regarding open service of trans service members have identified the need for strong leadership to communicate the benefits of an inclusive and diverse workforce, the development of written policy on all aspects of gender-affirming healthcare access and administrative processes to minimize impact on service member or unit readiness, robust education and training efforts, clear anti-harassment policies inclusive of trans people, and advisors to military leadership who have the knowledge, skills, and experience needed to provide evidence-based information. Equity-oriented monitoring is needed to continually examine the impact of military service policies on readiness and retention to support actionable, data-driven improvements to policies and their implementation.

## Data Availability

The datasets generated and/or analysed during the current study are not publicly available due data sharing regulations and requirements of the US Department of Defense. Requests for data must be made directly to the US Defense Health Agency by investigators through data sharing agreement procedures, as indicated here: https://health.mil/Military-Health-Topics/Privacy-and-Civil-Liberties/Data-Sharing-Agreements.

## Abbreviations

95% CI: 95% credibility interval
BEAST: Bayesian estimator of abrupt change, seasonality, and trend
DoD: Department of Defense
ICD: International Classification of Diseases
MCMC: Markov chain Monte Carlo
US: United States
